# Relationship between health-seeking behavior and digital health literacy in epilepsy patients

**DOI:** 10.1590/1980-220X-REEUSP-2025-0087en

**Published:** 2025-10-13

**Authors:** Emine Kaplan Serin, Derya Tülüce, Semra Usta

**Affiliations:** 1 Mersin University, Faculty of Nursing, Mersin, Turkey.; 2 Osmaniye Korkut Ata University, Faculty of Health Sciences, Osmaniye, Turkey.; 3 Mersin University, Institute of Health Sciences, Mersin, Turkey.

**Keywords:** Epilepsy, Nursing, Health Behavior, Digital Health, Health Literacy, Epilepsia, Enfermagem, Comportamentos Relacionados com a Saúde, Saúde Digital, Letramento em Saúde

## Abstract

**Objective:**

The present study examines the relationship between health-seeking behavior and digital health literacy in patients with epilepsy.

**Method:**

Included in this descriptive, cross-sectional study were 115 epilepsy patients diagnosed at the neurology outpatient clinic of a public hospital between July and November 2024. All participants completed the Digital Health Literacy Instrument (DHLI) and the Health-Seeking Behavior Scale (HLBS). Statistical analyses were conducted to assess correlations between the scales and to compare mean scores across different patient characteristics.

**Results:**

A weak but statistically significant correlation was observed between several sub-dimensions of the DHLI (including information seeking, evaluating reliability, determining relevance, content creation and privacy protection) and the total HLBS score (p < 0.05). When the average scores of DHLI and HLBS are compared with the level of education, seizure frequency and time spent on social media, the statistical difference between them is significant. Specifically, patients with lower educational attainment and limited social media use were found to have lower levels of digital health literacy.

**Conclusion:**

The findings of the present study suggest that an interactive relationship exists between digital health literacy and health-seeking behaviors among epilepsy patients. We recommend tailored educational interventions and professional healthcare support to ensure the dissemination of reliable digital health information. Future studies may explore this relationship further with the involvement of larger samples and alternative methodologies.

## RESUMO

**Objetivo:**

Este estudo investigou a relação entre o comportamento de busca por saúde e a alfabetização digital em saúde entre pacientes com epilepsia.

**Métodos:**

Trata-se de um estudo descritivo e transversal que incluiu 115 pacientes diagnosticados com epilepsia no ambulatório de neurologia de um hospital público entre julho e novembro de 2024. Todos os participantes responderam ao Instrumento de Alfabetização Digital em Saúde (DHLI) e à Escala de Comportamento de Busca por Saúde (HLBS). Foram realizadas análises estatísticas para avaliar correlações entre as escalas e comparar médias de escores segundo características sociodemográficas e clínicas.

**Resultados:**

Observou-se uma correlação fraca, porém estatisticamente significativa, entre diversas subdimensões do DHLI (busca de informações, avaliação da confiabilidade, determinação da relevância, criação de conteúdo e proteção da privacidade) e o escore total da HLBS (p < 0,05). As médias dos escores do DHLI e do HLBS variaram significativamente conforme o nível educacional, a frequência das crises e o tempo de uso das mídias sociais. Especificamente, pacientes com menor escolaridade e menor uso de redes sociais apresentaram níveis mais baixos de alfabetização digital em saúde.

**Conclusão:**

Os achados sugerem uma relação interativa entre a alfabetização digital em saúde e o comportamento de busca por saúde em pacientes com epilepsia. Recomenda-se a implementação de intervenções educacionais personalizadas e suporte profissional para promover o acesso a informações digitais confiáveis em saúde. Pesquisas futuras devem explorar essa relação com amostras maiores e metodologias diferenciadas.

## INTRODUCTION

Epilepsy is a chronic disorder of the central nervous system characterized by recurrent seizures resulting from abnormal electrical activity in the brain. Such seizures significantly affect the physical, mental and social life of people with epilepsy^(1)^. Approximately 70 million people worldwide have been diagnosed with epilepsy, revealing the global health burden of the disease^(2)^. Epilepsy can affect all age groups, and clinical symptoms may differ depending on the etiology^(3)^. The magnitude of untreated and uncontrolled epilepsy and epilepsy-related deaths in low- and middle-income countries reveals a need for urgent efforts to improve access to disease management^(4)^.

Epilepsy significantly reduces the quality of life of those affected due to the unexpected nature of seizures. The unpre-dictability of seizures and their disruptive impact on daily life lead patients to seek information about their disease as a health-seeking behavior, referring to any action or inaction taken by a person in an attempt to address a health problem or when she/he becomes ill^(1,3,5)^. Such actions can include visits to physicians, health professionals or health institution with health-related problems; self-medication, based on the recommendations of people in their close environment; and seeking information about the issue on the Internet, which has become particularly common^(5, 6, 7)^.

The evidence-based content found online can often be lost among the wealth of unreliable information, leading those diagnosed with epilepsy to be directed to inaccurate information. In this regard, careful Internet use is essential in disease management^(5,8, 9, 10)^. With the widespread use of the Internet and the adoption of mobile technologies, the general public has gained easy access to health-related information, leading to a growing awareness of disease management and health promotion^(9,11)^. People with epilepsy tend to look online for information on how to cope with the disease and improve their health, and advances in technology have made such behaviors more attractive and accessible.

Digital health literacy refers to the ability of the user to understand, evaluate and make use of online health information, helping people cope more effectively with their health problems and access appropriate health services^(5,9)^. The efforts of epilepsy patients to cope with their disease directly affect their health-seeking behaviors, and the role of digital health literacy in this process is becoming increasingly important. The present study makes use of two scales for the assessment of the respondents: the Digital Health Literacy Instrument (DHLI), which measures the ability of the respondent to use online health information; and the Health-Seeking Behavior Scale (HLBS), which assesses the health behaviors of the respondent. Both scales are theoretically appropriate and validated, and are thus deemed suitable for the study population^(12,13)^.

Studies examining the relationship between the health-seeking behaviors and digital health literacy of people with epilepsy are limited in Türkiye. There is, however, a wealth of international literature emphasizing the importance of digital health literacy for people with chronic conditions such as epilepsy, highlighting its contributions to health management and the availability of appropriate healthcare services^(6,9)^. Contributing to this body of literature, the present study offers an understanding of the health behaviors of epilepsy patients by raising the following questions among this patient group:

- What are the health-seeking behavior levels of epilepsy patients?
- What are the health literacy levels of epilepsy patients?
- Are the health-seeking behaviors and health literacy of epilepsy patients related?

It was hypothesized within the scope of this study that people with epilepsy with higher levels of digital health literacy will have more positive health-seeking behaviors.

## METHOD

### Study Design

This descriptive, cross-sectional study was conducted in a province in the southern region of Türkiye, and was conducted and reported in line with the STROBE guidelines for observational studies.

### Study Population

Included in the study were 115 patients who had been diagnosed with epilepsy in the neurology outpatient clinic of a public hospital between July and November 2024. All patients who met the following inclusion criteria were included in the study: aged 18 years or older, having received treatment for epilepsy for the last 1 month, and sufficiently literate to use social media. Excluded from the study were those with cognitive impairment, illiteracy, and such barriers to communication as impaired vision, hearing or speech.

### Measurement Tools

The study data were collected by the researchers using the “Descriptive Information Form”, the “Digital Health Literacy Scale (DHLI)” and the “Health Seeking Behavior Scale (HLBS)” during face-to-face interviews with those undergoing treatment for epilepsy. Data collection took approximately 30 minutes, and those collecting the information took care not to influence the responses of the participants. The interviews were conducted in a private, comfortable setting to ensure confidentiality and ethical compliance.

### Descriptive Information Form

The Descriptive Information Form included 19 questions, and was prepared by the researchers in light of the literature to garner data on the respondents’ age, sex, educational status, marital status, employment status, income status, presence of health insurance, smoking, seizure frequency, type of treatment received, type of seizure, most used social media networks and purpose of social media use^(5,12, 13, 14)^.

### Digital Health Literacy Scale (DHLI)

The Digital Health Literacy scale was developed by Van der Vaart and Constance Drosseart in 2017, and adapted into Turkish by Çetin and Gümüş (2023). The scale contains 18 items spread across six sub-dimensions, rated on a Likert-type scale. Among the sub-dimensions, Information Seeking, Assessing Reliability, Determining Level of Interest and Adding Content are coded as “4 = It is quite easy, 3 = It is easy, 2 = It is difficult, 1 = It is quite difficult”; the Navigation Skills and Privacy Protection sub-dimensions are coded as “Never = 4, Rarely = 3, Occasionally = 2 and Frequently = 1”; and the Navigation Skills and Privacy Protection sub-dimensions are reverse-coded. For the evaluation of the digital health literacy, the original developers of the scale assessed the mean scores from the scale and its sub-dimensions on a scale of 1–4, with mean scores lower than 2 considered “low”, between 2 and 3 considered “medium”, and greater than 3 considered “high”. The Cronbach alpha coefficient for the overall reliability of the original scale was 0.896^(12)^. In the present study, Cronbach’s alpha coefficient was 0.726.

### Health-Seeking Behavior Scale (HLBS)

The HLBS was developed by Kıraç and Öztürk in 2021, and includes 12 items across three subdimensions for the measurement of the health-seeking behaviors of individuals, with online health-seeking behaviors assessed with six items (items 1–6), professional health-seeking behavior assessed with three items (items 7–9) and traditional health-seeking behaviors assessed with three items (items 10–12). All scale items are positive and are rated on a 5-point Likert-type scale (1 = strongly disagree, 5 = strongly agree). None of the items are scored in reverse. Total scores from the scale are in the range of 12 to 60. The Cronbach’s Alpha coefficients of the original study indicating the reliability of the scale, were 0.726 for online health seeking, 0.720 for professional health seeking, 0.736 for traditional health seeking and 0.755 for the overall scale. In the present study, Cronbach’s alpha coefficients were 0.800 for online health seeking, 0.713 for professional health seeking, 0.685 for the traditional health seeking and 0.724 for the overall scale^(13)^.

### Research Variables

The dependent variable in the study was the mean score from the digital health literacy scale, while the independent variable was the mean score from the patients’ sociodemographic characteristics, disease-related characteristics and health-seeking behavior scale score.

### Data Analysis

The study data were analyzed using the SPSS 25.0 program. Number, percentage, mean and standard deviation analyses were used for the evaluation of descriptive data. Student t-tests were used to analyze bivariate data, and an ANOVA was used to analyze data with three or more variables. Pearson’s correlation was used to assess the relationship between the mean scores of the scales.

### Ethical Considerations

Written informed consent was obtained from the head physician of the hospital in which the study was conducted as well as from the relevant provincial health directorate, with registration numbers E.181509 dated 07.06.2024, and E-249914660 dated 30.07.2024. Before starting the study, all those included gave written and verbal permission for the use of their data. The study was conducted in accordance with the principles agreed upon in the Declaration of Helsinki.

## RESULTS

The mean age of the patients was 38.36 ± 16.51 (18–85) years, 54.8% were male, 65.2% were married, 47.8% were high school graduates, 70.4% were unemployed, 80.0% had health insurance, 69.6% lived in the city center and 93.9% had a nuclear family structure (Table 1).

**Table 1:** Sociodemographic characteristics of the patients – Osmaniye, Türkiye, 2024.

Features	% (n)
Gender	
Woman	45.2 (52)
Male	54.8 (63)
Marital status	
Married	65.2 (75)
Single	34.8 (40)
Education status	
Illiterate	9.6 (11)
Literate	6.1 (7)
Primary school graduate	25.2 (29)
High school graduate	47.8 (55)
University and above	11.3 (13)
Employment status	
Working	29.6 (34)
Not working	70.4 (81)
Income status	
Income less than expenditure	42.6 (49)
Income equal to expenditure	51.3 (59)
Income more than expenditure	6.1 (7)
Health insurance	
There is	80.0 (92)
No	20.0 (23)
Place of residence	
Province	69.6 (80)
District	22.6 (26)
Village	7.8 (9)
Family structure	
Nuclear family	93.9 (108)
Extended family	6.1 (7)

It was determined that 55.7% of the patients had experienced generalized seizures, 71.3% received a single treatment, 87.0% used regular medication, 45.2% experienced day and night seizures, 71.3% did not smoke and 45.2% experienced more than one seizure per month (Table 2).

**Table 2:** Disease characteristics of the patients – Osmaniye, Türkiye, 2024.

Features	% (n)
Seizure type	
Generalized	55.7 (64)
Tonic	19.1 (22)
Clonic	6.1 (7)
Simple partial	8.7 (10)
Complex partial	5.2 (6)
Absenteeism	0.9 (1)
Myoclonic	4.3 (5)
Treatment Received	
Single	71.3 (82)
Multiple	28.7 (33)
Medication use status	
Regular	87.0 (100)
Irregular	13.0 (15)
Watch time	
Day	29.6 (34)
Night	25.2 (29)
Day-night	45.2 (52)
Smoking status	
Yes	28.7 (33)
No	71.3 (82)
Seizure frequency	
Once a month	40.9 (47)
More than once a month	45.2 (52)
Nothing	13.9 (16)

Some 40.0% of the respondents used social media for 2–3 hours a day, 43.5% used Facebook, 14.8% used X, 43.5% used Instagram and 47.0% used social media as usual. The purposes of using social media were to utilize leisure time (57.4%), access information (35.7%), reach people (35.7%), exchange messages (21.7%), listen to music (8.9%), share presentations and information (5.2%), and get to know people better (5.2%), respectively (Table 3).

**Table 3:** Characteristics of patients' social media use – Osmaniye, Türkiye, 2024.

Features	% (n)
Time of social media use	
1 hour or less	39.1 (45)
2-3 hours	40.0 (46)
4-5 hours	7.8 (9)
6 hours or more	13.0 (15)
Facebook	
Yes	43.5 (50)
No	56.5 (65)
X	
Yes	14.8 (17)
No	85.2 (98)
Instagram	
Yes	43.5 (50)
No	56.5 (65)
Social media time change	
Fewer	43.5 (50)
As much as ever	47.0 (54)
More than ever	6.1 (7)
More than ever before	3.5 (4)

The mean scores of the respondents from the digital health literacy scale sub-dimensions were 2.61 ± 0.88 information seeking, 2.37 ± 0.79 assessing reliability, 2.57 ± 1.04 determining level of interest, 2.71 ± 0.79 adding content, 1.48 ± 0.72 navigation skills, 1.70 ± 0.45 protection of privacy with a total mean score of 2.23 ± 0.37. The mean scores from the health- seeking behaviors scale sub-dimension, on the other hand, were 14.46 ± 6.76 for online health seeking, 13.19 ± 2.88 for professional health seeking and 10.07 ± 3.61 for traditional health seeking, with a total mean score of 37.73 ± 8.90 (Table 4).

**Table 4:** Total and sub-dimension score averages of the digital health literacy scale and health-seeking behaviors scale – Osmaniye, Turquía, 2024.

Sub-Scales	Mean ± sd
Information Seeking	2.61 ± 0.88
Assessing Reliability	2.37 ± 0.79
Determining Level of Interest	2.57 ± 1.04
Adding Content	2.71 ± 0.79
Navigation Skills	1.48 ± 0.72
Protection of Privacy	1.70 ± 0.45
Digital Health Literacy Instrument	2.23 ± 0.37
Online Health Seeking	14.46 ± 6.76
Professional Health Seeking	13.19 ± 2.88
Traditional Health Seeking	10.07 ± 3.61
Health-Seeking Behaviors Scale	37.73 ± 8.90

An analysis of the relationship between the digital health literacy scale and its sub-dimensions, and the health-seeking behaviors scale and its sub-dimensions revealed a weak but statistically significant relationship between the OSH’s digital health literacy scale sub-dimensions related to information seeking, evaluation of reliability, determining the level of interest, adding content, protection of privacy and scale total score. Furthermore, a weak but statistically significant relationship was found between the “assessment of reliability” sub-dimension of the digital health literacy scale and professional health-seeking behavior (p < .05). Furthermore, a statistically significant but weak relationship was noted between information seeking, evaluation of reliability, determining the level of interest and total scale score sub-dimensions of the DHLI (Table 5).

**Table 5:** Correlation between digital health literacy scale and its sub-dimensions and health-seeking behaviors scale and its sub-dimensions – Osmaniye, Türkiye, 2024.

	OSH		PHS		THS		HLBS	
	r	p	r	p	r	p	r	p
IS	,378**	**<0.001**	,099	0.291	,022	0.812	,329**	**<0.001**
AR	,296**	**0.001**	,211*	**0.024**	,093	0.323	,331**	**<0.001**
DLI	**,392**	**<0.001**	,180	0.055	,081	0.388	**0.389**	**<0.001**
NS	-,118	0.210	-,131	0.163	-,066	0.481	-,159	0.090
AC	,297*	**0.001**	-,010	0.919	-,174	0.063	,152	0.104
PP	−,218*	**0.019**	,087	0.358	,100	0.286	-,097	0.302
DHLI	,401**	**<0.001**	,149	0.111	,064	0.495	,379**	**<0.001**

IS – Information Seeking; AR – Assessing Reliability; DLI – Determining Level of Interest; AC – Adding Content; NS – Navigation Skills; PP – Protection of Privacy; DHLI – Digital Health Literacy Instrument; OSH – Online Health Seeking; PHS – Professional Health Seeking; THS – Traditional Health Seeking; HLBS – Health Seeking Behaviors Scale; r – Pearson’s correlation coefficient.

*p < 0.05

**p < 0.001.

**Figure 1:**
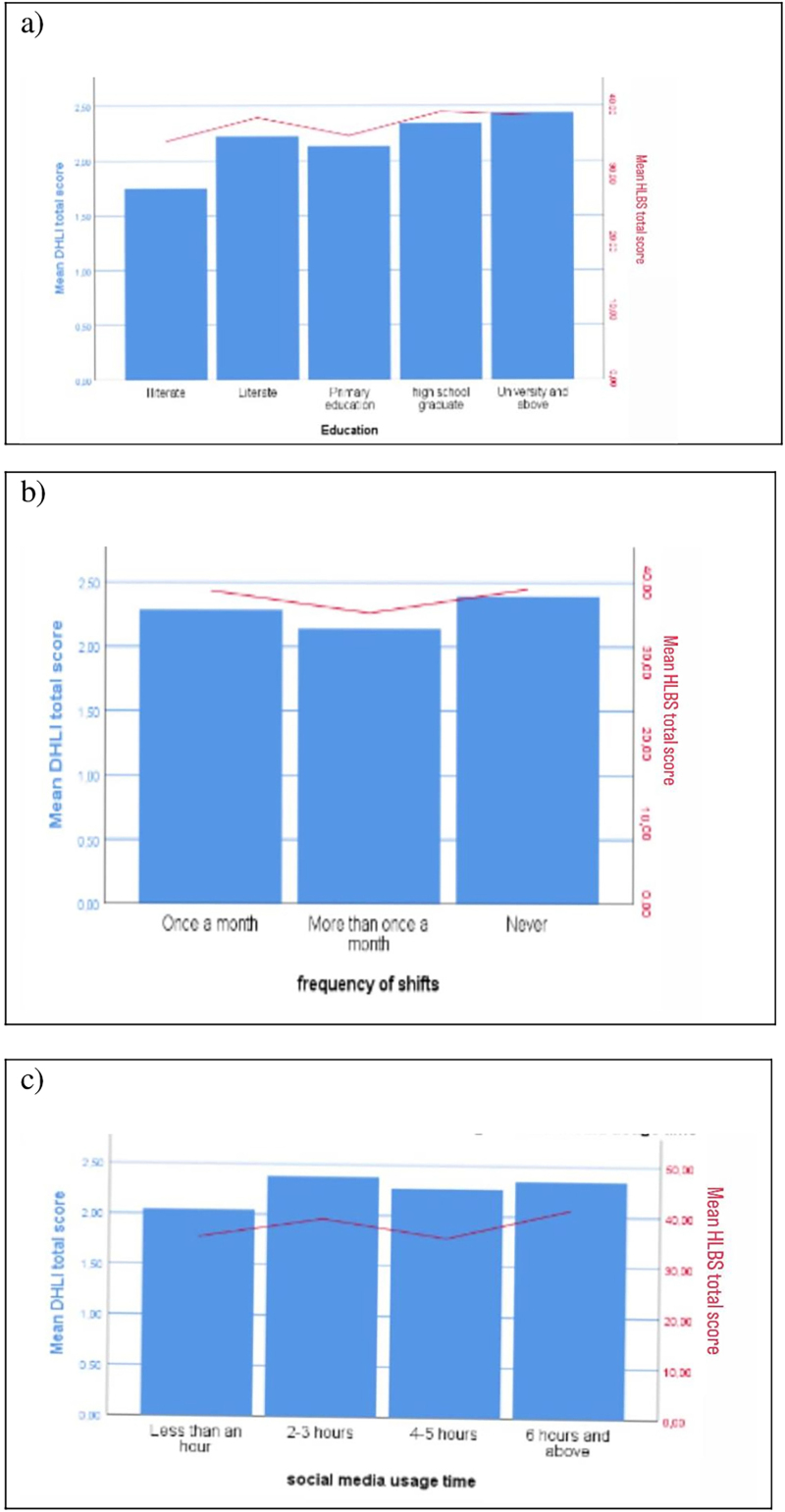
Change in DHLI and HLBS mean scores according to educational status, seizure frequency, and social media use time.

The respondents’ sociodemographic data, disease status and social media use characteristics were analyzed to identify any differences between the mean scores of the HLBS and DHLI, revealing significant differences in educational status, seizure frequency and social media use duration (p < 0.05) (Figure 1).

The mean DHLI score was compared with that of the educational level scale, revealing the mean score of the illiterate group to be statistically lower than that of the other education groups. A subsequent comparison of the mean Digital Health Literacy Instrument (DHLI) scores based on shift frequency revealed that participants who undertook a few shifts per month had significantly lower mean scores compared to those who did not work any shifts (p < .05). A comparison of the mean score from the DHLI with the time spent on social media revealed that those who used social media for 1 hour or less a day had a statistically lower mean score than those who used it for 2–3 hours and 6 hours or more a day.

## DISCUSSION

The findings of the present study make an essential contribution to knowledge of the relationship between the health-seeking behaviors and digital health literacy among epilepsy patients, revealing the effects of various factors. The results suggest that sociodemographic characteristics, disease status and social media may have a significant effect on the health-seeking behaviors and digital health literacy of this patient group.

### Sociodemographic Characteristics and Digital Health Literacy

It was noted that most of the participants were male, married, high school graduates, unemployed and residents of the city center, and education level had a significant effect on digital health literacy and health-seeking behaviors. In this regard, the study supports the findings of previous studies reporting an association between high digital health literacy levels and health information-seeking behaviors over the Internet among those with higher education levels^(9,15)^. In the present study, finding significant differences between education level and digital health literacy and health-seeking behavior is consistent with similar findings. In the literature, It has been observed that as education level increases, people become more aware of how to evaluate the reliability of digital health information. In a study of 200 older adult patients diagnosed with Parkinson’s, 73% of the sample with a low income status were found to have a low level of digital health literacy^(16)^. In a further study of older adults, 84.9% of the respondents stated they had difficulty accessing digital health services, and an average e-health literacy scale score of 18.43 ± 10 was recorded for the sample^(17)^. In an earlier study of epilepsy patients, 38.1% were single and 8.4% were unemployed, and a significant negative correlation was observed between rural residency (p < 0.001), unemployment (p = 0.006) and low educational level among the patients (p < 0.001)^(18)^.

### Illness Status and Health-Seeking Behavior

The fact that most epilepsy patients take regular medication and have generalized seizures is another crucial factor affecting health-seeking behaviors. People living with chronic diseases such as epilepsy have been found to seek more information about disease management^(10,11)^. In the present study, the majority of the respondents turned to the Internet for health- related information and received regular treatment. There are concerns, however, regarding the frequency at which patients look for health information, and the reliability of the material they encounter. Health information accessed via the Internet varies widely in terms of accuracy and reliability^(8)^, and so the importance of digital health literacy should not be underrated. Patients with low levels of digital health literacy are in danger of being misled by erroneous information when looking for health information online. In a study conducted during the COVID-19 pandemic, a 1-point increase in access to health information about COVID-19 was associated with a 3.01-point increase in general digital health literacy, revealing the extent to which people engage in health-seeking behaviors when they fall ill^(19)^.

### Social Media Use and Digital Health Literacy

The effects of social media on health-seeking behaviors were also assessed in the present study. A large proportion of patients turn to social media for information. Previous studies in the literature report social media to be an essential source of health information^(20, 21, 22)^. It should be noted, however, that information obtained through social media may be misleading for people with low health literacy levels^(23,24)^. In the present study an association was found between the use of social media to obtain information, and both digital health literacy and health-seeking behaviors. However, the safety of social media as a source of information related to health depends on the digital health literacy level of the individual. In a study of the reasons why people use the Internet, social media ranked first, with 30.5%, followed by video calls with 25.3% and as a source of health-related data with 20.2%^(17)^. In an earlier study of epilepsy patients, 42.6% reported being aware of digital health applications^(25)^.

### Relationship Between Digital Health Literacy and Health-Seeking Behaviors

Our findings reveal a weak but significant relationship between health literacy and health-seeking behaviors. In particular, the “searching for information”, “evaluating credibility” and “adding content” sub-dimensions of digital health literacy are associated with health-seeking behaviors. It has been previously reported people with high digital health literacy levels are better able to evaluate the health information they find through the Internet, and to act more consciously when applying to health professionals^(9)^. In contrast, the lack of a significant relationship between the “privacy protection” and “navigation skills” sub-dimensions of digital health literacy and health-seeking behavior suggests that the difficulties encountered while searching for health information in the digital environment are not directly related to these skills. In a study conducted in Europe, it was reported that 71% of the respondents used the Internet for health purposes, and that such habits were most prominent among the young and those with higher education levels, while the number of visits to general practitioners in the year, long-term illness or disability, and good subjective health assessment also had a positive effect^(26)^. According to Eurostat, in 2020, 55% of the respondents in a study of 27 European countries were found to use the Internet to search for health information^(27)^. It reveals that socioeconomic factors such as education and income level affect the ability of individuals to access and use digital tools. Indeed, in this study, a lower education level and lower income were found to be significantly associated with lower health literacy (p < 0.001)^(28)^. Digital health literacy depends not only on the skills of the individual, but also the accessibility and design of digital platforms. In a study conducted in Türkiye, the respondents diagnosed with epilepsy were found to have a medium level of health literacy, and those with higher levels of health literacy had more positive attitudes towards their condition^(29)^. A direct association between low education and income levels and health literacy levels has been reported in the literature, while higher education levels have been linked to decreased seizure frequencies and emergency department visits, though it has been stressed that there is no direct association between health literacy and such clinical outcomes^(28)^. It is predicted that digital platforms, drug information materials, labels, patient brochures, educational staff support, and content summaries presented in plain and understandable language and developed to increase epilepsy-specific health literacy, can contribute to improving the general health status of individuals by facilitating their access to health information^(30)^. This paper suggests that disseminating information that is both accessible and understandable can indirectly improve the health literacy of people with epilepsy, as well as their health and their attitudes towards their condition. Furthermore, investing in health literacy can play a strategic role in strengthening the control of patients over their health.

This study has revealed a two-way relationship between the health-seeking behaviors of epilepsy and their digital health literacy levels. Educational and disease status, as well as social media use, all contribute to digital health literacy and the development of health-seeking behaviors.

### Limitations

The limitations of the present study include its single-center study design, its small sample size and its use of a questionnaire for the collection of data. Furthermore, the sample included only patients who presented to hospital, and so contacting a larger patient group was not possible. Future studies can examine this issue in greater depth through the involvement of a larger sample and different research methods.

## CONCLUSION

This study has revealed a weak but significant association between digital health literacy and healthcare seeking behaviors in patients with epilepsy, with a low education level, high income and limited social media use being associated with reduced digital health literacy levels and healthcare seeking habits. These findings suggest that there is a need for targeted education programs aimed at increasing digital health literacy in this patient group, and for healthcare professionals to be trained on how to contribute to the availability of accurate and trustworthy health information in the digital environment. If healthcare professionals can be directed to share accurate health information with digital aging data, access to accurate patient characteristics will be facilitated.

## Data Availability

The data that support the findings of this study are available from the corresponding author upon reasonable request.
