# Supplementary material for: Relationship between health-seeking behavior and digital health literacy in epilepsy patients
Source: Rev Esc Enferm USP. 2025 Oct 13;59:e20250087. doi: 10.1590/1980-220X-REEUSP-2025-0087en (PMC12517545; doi:10.1590/1980-220X-REEUSP-2025-0087en)
Supplement: Translation

# Translation: Relación entre el comportamiento orientado a la búsqueda de atención sanitaria y la alfabetización digital en materia de salud en pacientes con epilepsia

**DOI:** 10.1590/1980-220X-REEUSP-2025-0087es

**Authors:** Emine Kaplan Serin, Derya Tülüce, Semra Usta

**Affiliations:** 1 Mersin University, Faculty of Nursing, Mersin, Turkey.; 2 Osmaniye Korkut Ata University, Faculty of Health Sciences, Osmaniye, Turkey.; 3 Mersin University, Institute of Health Sciences, Mersin, Turkey.

**Keywords:** Epilepsia, Enfermería, Conductas Relacionadas con la Salud, Salud Digital, Alfabetización en Salud

## Abstract

**Objetivo:**

Este estudio analiza la relación entre la conducta de búsqueda de salud y la alfabetización digital en salud en pacientes con epilepsia.

**Método:**

Se llevó a cabo un estudio descriptivo y transversal con la participación de 115 pacientes diagnosticados con epilepsia en la consulta externa de neurología de un hospital público entre julio y noviembre de 2024. Todos los participantes completaron el Instrumento de Alfabetización Digital en Salud (DHLI) y la Escala de Conducta de Búsqueda de Salud (HLBS). Se realizaron análisis estadísticos para evaluar las correlaciones entre ambas escalas y comparar las puntuaciones medias según las características sociodemográficas y clínicas.

**Resultados:**

Se encontró una correlación débil pero estadísticamente significativa entre varias subdimensiones del DHLI (búsqueda de información, evaluación de la fiabilidad, determinación de la relevancia, creación de contenido y protección de la privacidad) y la puntuación total de la HLBS (p < 0,05). Las puntuaciones medias del DHLI y de la HLBS variaron significativamente en función del nivel educativo, la frecuencia de crisis epilépticas y el tiempo dedicado a redes sociales. En particular, los pacientes con menor nivel educativo y menor uso de redes sociales presentaron niveles más bajos de alfabetización digital en salud.

**Conclusión:**

Los resultados sugieren que existe una relación interactiva entre la alfabetización digital en salud y las conductas de búsqueda de salud en pacientes con epilepsia. Se recomienda implementar intervenciones educativas personalizadas y brindar apoyo profesional para facilitar el acceso a información digital confiable en salud. Futuras investigaciones deberían explorar esta relación con muestras más amplias y enfoques metodológicos alternativos.

## INTRODUCCIÓN

La epilepsia es un trastorno crónico del sistema nervioso central que se caracteriza por convulsiones recurrentes como resultado de una actividad eléctrica anormal en el cerebro. Estas crisis convulsivas afectan significativamente la vida física, mental y social de las personas con epilepsia^(1)^. Aproximadamente 70 millones de personas en todo el mundo han sido diagnosticadas con epilepsia, lo que revela la carga sanitaria mundial que supone esta enfermedad^(2)^. La epilepsia puede afectar a todos los grupos de edad y los síntomas clínicos pueden variar en función de la etiología^(3)^. La magnitud de la epilepsia no tratada y no controlada y de las muertes relacionadas con la epilepsia en los países de ingresos bajos y medios pone de manifiesto la necesidad de adoptar medidas urgentes para mejorar el acceso al tratamiento de la enfermedad^(4)^.

La epilepsia reduce significativamente la calidad de vida de las personas afectadas debido a la naturaleza impredecible de las convulsiones. La imprevisibilidad de las convulsiones y su impacto disruptivo en la vida cotidiana llevan a los pacientes a buscar información sobre su enfermedad como un comportamiento de búsqueda de salud, refiriéndose a cualquier acción o inacción tomada por una persona en un intento de abordar un problema de salud o cuando se enferma^(1,3,5)^. Estas acciones pueden incluir visitas a médicos, profesionales de la salud o instituciones sanitarias con problemas relacionados con la salud; la automedicación, basada en las recomendaciones de personas de su entorno cercano; y la búsqueda de información sobre el tema en Internet, lo que se ha vuelto particularmente común^(5, 6, 7)^.

El contenido basado en la evidencia que se encuentra en línea a menudo puede perderse entre la gran cantidad de información poco fiable, lo que lleva a las personas diagnosticadas con epilepsia a obtener información inexacta. En este sentido, es esencial un uso cuidadoso de Internet en el manejo de la enfermedad^(5,8, 9, 10)^. Con el uso generalizado de Internet y la adopción de las tecnologías móviles, el público en general ha obtenido un fácil acceso a la información relacionada con la salud, lo que ha llevado a una mayor concienciación sobre el manejo de las enfermedades y la promoción de la salud^(9,11)^. Las personas con epilepsia tienden a buscar en Internet información sobre cómo hacer frente a la enfermedad y mejorar su salud, y los avances tecnológicos han hecho que estos comportamientos sean más atractivos y accesibles.

La alfabetización sanitaria digital (Digital Health Literacy en inglés) se refiere a la capacidad del usuario para comprender, evaluar y utilizar la información sanitaria en línea, lo que ayuda a las personas a afrontar con mayor eficacia sus problemas de salud y a acceder a los servicios sanitarios adecuados^(5,9)^. Los esfuerzos de los pacientes con epilepsia para afrontar su enfermedad afectan directamente a sus comportamientos de búsqueda de la salud, y el papel de la alfabetización sanitaria digital en este proceso es cada vez más importante. El presente estudio utiliza dos escalas para la evaluación de los encuestados: el Instrumento de Alfabetización Digital en Salud (Digital Health Literacy Instrument – DHLI), que mide la capacidad del encuestado para utilizar la información sanitaria en línea; y la Escala de Comportamientos de Búsqueda de Salud (Health-Seeking Behavior Scale – HLBS), que evalúa los comportamientos de salud del encuestado. Ambas escalas son adecuadas y validadas por la teoría, por lo que se consideran adecuadas para la población del estudio^(12,13)^.

Los estudios que examinan la relación entre los comportamientos de búsqueda de atención sanitaria y la alfabetización sanitaria digital de las personas con epilepsia son limitados en Turquía. Sin embargo, existe una abundante bibliografía internacional que destaca la importancia de la alfabetización sanitaria digital para las personas con enfermedades crónicas como la epilepsia, y subraya su contribución a la gestión de la salud y a la disponibilidad de servicios sanitarios adecuados^(6,9)^. Contribuyendo a este conjunto de publicaciones, el presente estudio ofrece una comprensión de los comportamientos de salud de los pacientes con epilepsia planteando las siguientes preguntas entre este grupo de pacientes:

- ¿Cuáles son los niveles de comportamiento de búsqueda de atención sanitaria de los pacientes con epilepsia?
- ¿Cuáles son los niveles de alfabetización sanitaria de los pacientes con epilepsia?
- ¿Están relacionados los comportamientos de búsqueda de atención sanitaria y la alfabetización sanitaria de los pacientes con epilepsia?

En el ámbito de este estudio, se planteó la hipótesis de que las personas con epilepsia con niveles más altos de alfabetización sanitaria digital tendrán comportamientos más positivos en la búsqueda de la salud.

## MÉTODO

### Diseño del Estudio

Este estudio descriptivo y transversal se llevó a cabo en una provincia de la región sur de Turquía, y se realizó y documentó de acuerdo con las directrices STROBE para estudios observacionales.

### Población del Estudio

Se incluyeron en el estudio 115 pacientes que habían sido diagnosticados con epilepsia en la consulta externa de neurología de un hospital público entre julio y noviembre de 2024. Se incluyeron en el estudio todos los pacientes que cumplían los siguientes criterios de inclusión: edad igual o superior a 18 años, haber recibido tratamiento para la epilepsia durante el último mes y tener un nivel de alfabetización suficiente para utilizar las redes sociales. Se excluyó del estudio a aquellos con deterioro cognitivo, analfabetismo y barreras de comunicación tales como discapacidad visual, auditiva o del habla.

### Herramientas de Medición

Los datos del estudio fueron recolectados por los investigadores utilizando el Formulario de información descriptiva, el Instrumento de alfabetización digital en salud (DHLI) y la Escala de comportamiento de búsqueda de salud (HLBS) durante entrevistas cara a cara con las personas que estaban recibiendo tratamiento para la epilepsia. La recopilación de datos duró aproximadamente 30 minutos, y quienes recolectaron la información tuvieron cuidado de no influir en las respuestas de los participantes. Las entrevistas se realizaron en un entorno privado y cómodo para garantizar la confidencialidad y el cumplimiento ético.

### Formulario de Información Descriptiva

El formulario de información descriptiva incluía 19 preguntas y fue elaborado por los investigadores a partir de la bibliografía disponible para recabar datos sobre la edad, el sexo, el nivel educativo, el estado civil, la situación laboral, los ingresos, la existencia de seguro médico, el tabaquismo, la frecuencia de las crisis, el tipo de tratamiento recibido, el tipo de crisis, las redes sociales más utilizadas y el propósito del uso de las redes sociales de los encuestados^(5,12, 13, 14)^.

### Escala de Alfabetización Digital en Salud (DHLI)

La escala de alfabetización digital en salud fue desarrollada por Van der Vaart y Constance Drosseart en 2017 y adaptada al turco por Çetin y Gümüş (2023). La escala contiene 18 ítems distribuidos en seis subdimensiones, calificados en una escala tipo Likert. Entre las subdimensiones, la búsqueda de información, la evaluación de la fiabilidad, la determinación del nivel de interés y la adición de contenido se codifican como «4 = Es bastante fácil, 3 = Es fácil, 2 = Es difícil, 1 = Es bastante difícil»; las subdimensiones «Habilidades de navegación» y «Protección de la privacidad» se codifican como «Nunca = 4, Rara vez = 3, Ocasionalmente = 2 y Frecuentemente = 1»; y las subdimensiones «Habilidades de navegación» y «Protección de la privacidad» se codifican a la inversa. Para la evaluación de la alfabetización digital en salud, los desarrolladores originales de la escala evaluaron las puntuaciones medias de la escala y sus subdimensiones en una escala de 1 a 4, considerando las puntuaciones medias inferiores a 2 como «bajas», entre 2 y 3 como «medias» y superiores a 3 como «altas». El coeficiente alfa de Cronbach para la fiabilidad global de la escala original fue de 0,896.^(12)^ En el presente estudio, el coeficiente alfa de Cronbach fue de 0,726.

### Escala de Comportamientos de Búsqueda de Salud (HLBS)

La HLBS fue desarrollada por Kıraç y Öztürk en 2021 e incluye 12 ítems en tres subdimensiones para la medición de los comportamientos de búsqueda de salud de las personas, con los comportamientos de búsqueda de salud en línea evaluados con seis ítems (ítems 1–6), el comportamiento de búsqueda de salud profesional se evalúa con tres ítems (ítems 7–9) y los comportamientos de búsqueda de salud tradicionales se evalúan con tres ítems (ítems 10–12). Todos los ítems de la escala son positivos y se califican en una escala tipo Likert de 5 puntos (1 = totalmente en desacuerdo, 5 = totalmente de acuerdo). Ninguno de los ítems se puntúa a la inversa. Las puntuaciones totales de la escala oscilan entre 12 y 60. Los coeficientes alfa de Cronbach del estudio original, que indican la fiabilidad de la escala, fueron de 0,726 para la búsqueda de información sanitaria en línea, 0,720 para la búsqueda de información sanitaria profesional, 0,736 para la búsqueda de información sanitaria tradicional y 0,755 para la escala global. En el presente estudio, los coeficientes alfa de Cronbach fueron de 0,800 para la búsqueda de información sanitaria en línea, 0,713 para la búsqueda de información sanitaria profesional, 0,685 para la búsqueda de información sanitaria tradicional y 0,724 para la escala global^(13)^.

### Variables de Investigación

La variable dependiente del estudio fue la puntuación media de la escala de alfabetización sanitaria digital, mientras que la variable independiente fue la puntuación media de las características sociodemográficas de los pacientes, las características relacionadas con la enfermedad y la puntuación de la escala de comportamiento de búsqueda de información sanitaria.

### Análisis de Datos

Los datos del estudio se analizaron utilizando el programa SPSS 25.0. Se utilizaron análisis de número, porcentaje, media y desviación estándar para la evaluación de los datos descriptivos. Se utilizaron pruebas t de Student para analizar los datos bivariados y un ANOVA para analizar los datos con tres o más variables. Se utilizó la correlación de Pearson para evaluar la relación entre las puntuaciones medias de las escalas.

### Consideraciones Éticas

Se obtuvo el consentimiento informado por escrito del médico jefe del hospital en el que se realizó el estudio, así como de la dirección provincial de salud correspondiente, con los números de registro E.181509 de fecha 07.06.2024 y E-249914660 de fecha 30.07.2024. Antes de iniciar el estudio, todos los participantes dieron su consentimiento por escrito y verbal para el uso de sus datos. El estudio se llevó a cabo de acuerdo con los principios acordados en la Declaración de Helsinki.

## RESULTADOS

La edad promedio de los pacientes era de 38,36 ± 16,51 (18-85) años, el 54,8% eran hombres, el 65,2% estaban casados, el 47,8% tenían estudios secundarios, el 70,4% estaban desempleados, el 80,0% tenían seguro médico, el 69,6% vivían en el centro de la ciudad y el 93,9% tenían una estructura familiar nuclear (Tabla 1).

**Tabla 1:** Características sociodemográficas de los pacientes – Osmaniye, Turquía, 2024.

Características	% (n)
Género	
Mujer	45,2 (52)
Hombre	54,8 (63)
Estado civil	
Casado	65,2 (75)
Soltero	34,8 (40)
Nivel educativo	
Analfabeto	9,6 (11)
No analfabeto	6,1 (7)
Graduado de primaria	25,2 (29)
Graduado de secundaria	47,8 (55)
Universitario y superior	11,3 (13)
Situación laboral	
Trabajando	29,6 (34)
Sin empleo	70,4 (81)
Situación de ingresos	
Ingresos inferiores a los gastos	42,6 (49)
Ingresos iguales a los gastos	51,3 (59)
Ingresos superiores a los gastos	6,1 (7)
Seguro médico	
Sí	80,0 (92)
No	20,0 (23)
Lugar de residencia	
Provincia	69,6 (80)
Distrito	22,6 (26)
Pueblo	7,8 (9)
Estructura familiar	
Familia nuclear	93,9 (108)
Familia extensa	6,1 (7)

Se determinó que el 55,7% de los pacientes había experimentado convulsiones generalizadas, el 71,3% recibió un único tratamiento, el 87,0% utilizaba medicación habitual, el 45,2% experimentaba convulsiones diurnas y nocturnas, el 71,3% no fumaba y el 45,2% experimentaba más de una convulsión al mes (Tabla 2).

**Tabla 2:** Características de la enfermedad de los pacientes – Osmaniye, Turquía, 2024.

Características	% (n)
Tipo de crisis convulsiva	
Generalizadas	55,7 (64)
Tónico	19,1 (22)
Clónica	6,1 (7)
Parcial simple	8,7 (10)
Parcial compleja	5,2 (6)
Ausentismo	0,9 (1)
Mioclónico	4,3 (5)
Tratamiento recibido	
Único	71,3 (82)
Múltiple	28,7 (33)
Uso de medicación	
Regular	87,0 (100)
Irregular	13,0 (15)
Tiempo de observación	
Días	29,6 (34)
Noche	25,2 (29)
Día-noche	45,2 (52)
Tabaquismo	
Sí	28,7 (33)
No	71,3 (82)
Frecuencia de las convulsiones	
Una vez al mes	40,9 (47)
Más de una vez al mes	45,2 (52)
Ninguna	13,9 (16)

Alrededor del 40,0% de los encuestados utilizaba las redes sociales entre 2 y 3 horas al día, el 43,5% utilizaba Facebook, el 14,8% utilizaba X, el 43,5% utilizaba Instagram y el 47,0% utilizaba las redes sociales como de costumbre. Los motivos para utilizar las redes sociales eran aprovechar el tiempo libre (57,4%), acceder a información (35,7%), contactar con personas (35,7%), intercambiar mensajes (21,7%), escuchar música (8,9%), compartir presentaciones e información (5,2%) y conocer mejor a otras personas (5,2%), respectivamente (Tabla 3).

**Tabla 3:** Características del uso de las redes sociales por parte de los pacientes – Osmaniye, Turquía, 2024.

Características	% (n)
Tiempo de uso de las redes sociales	
1 hora o menos	39,1 (45)
2-3 horas	40,0 (46)
4-5 horas	7,8 (9)
6 horas o más	13,0 (15)
Facebook	
Sí	43,5 (50)
No	56,5 (65)
X	
Sí	14,8 (17)
No	85,2 (98)
Instagram	
Sí	43,5 (50)
No	56,5 (65)
Cambio en el tiempo dedicado a las redes sociales	
Menos	43,5 (50)
Igual que siempre	47,0 (54)
Más que nunca	6,1 (7)
Más que nunca	3,5 (4)

Las puntuaciones medias de los encuestados en las subdimensiones de la escala de alfabetización sanitaria digital fueron 2,61 ± 0,88 (búsqueda de información), 2,37 ± 0,79 en búsqueda de información, 2,57 ± 1,04 en evaluación de la fiabilidad, 2,71 ± 0,79 en determinación del nivel de interés, 2,71 ± 0,79 en adición de contenido, 1,48 ± 0,72 en habilidades de navegación y 1,70 ± 0,45 en protección de la privacidad, con una puntuación media total de 2,23 ± 0,37. Por otro lado, las puntuaciones medias de la subdimensión de la escala de comportamientos de búsqueda de salud fueron 14,46 ± 6,76 para la búsqueda de salud en línea, 13,19 ± 2,88 para la búsqueda de salud profesional y 10,07 ± 3,61 para la búsqueda de salud tradicional, con una puntuación media total de 37,73 ± 8,90 (Tabla 4).

**Tabla 4:** Promedios de las puntuaciones totales y de las subdimensiones de la escala de alfabetización digital en salud y la escala de comportamientos de búsqueda de salud – Osmaniye, Turquía, 2024.

Subescalas	Media ± sd
Búsqueda de información	2,61 ± 0,88
Evaluación de la fiabilidad	2,37 ± 0,79
Determinación del nivel de interés	2,57 ± 1,04
Añadir contenido	2,71 ± 0,79
Habilidades de navegación	1,48 ± 0,72
Protección de la privacidad	1,70 ± 0,45
Alfabetización sanitaria digital Instrumento	2,23 ± 0,37
Búsqueda de información sanitaria en línea	14,46 ± 6,76
Búsqueda profesional de información sobre salud	13,19 ± 2,88
Búsqueda tradicional de información sobre salud	10,07 ± 3,61
Escala de comportamientos orientados a la salud	37,73 ± 8,90

Un análisis de la relación entre la escala de alfabetización digital en salud y sus subdimensiones, y la escala de conductas orientadas a la salud y sus subdimensiones reveló una relación débil pero estadísticamente significativa entre las subdimensiones de la escala de alfabetización digital en salud de la OSH relacionadas con la búsqueda de información, la evaluación de la fiabilidad, la determinación del nivel de interés, la adición de contenido, la protección de la privacidad y la puntuación total de la escala. Además, se encontró una relación débil pero estadísticamente significativa entre la subdimensión «evaluación de la fiabilidad» de la escala de alfabetización digital en salud y el comportamiento profesional de búsqueda de salud (p < 0,05). Además, se observó una relación estadísticamente significativa pero débil entre la búsqueda de información, la evaluación de la fiabilidad, la determinación del nivel de interés y la puntuación total de la escala de la DHLI (Tabla 5).

**Tabla 5:** Correlación entre la escala de alfabetización digital en salud y sus subdimensiones y la escala de comportamientos de búsqueda de salud y sus subdimensiones – Osmaniye, Turquía, 2024.

	OSH		PHS		THS		HLBS	
	r	p	r	p	r	p	r	p
IS	,378**	**<0,001**	,099	0,291	,022	0,812	,329**	**<0,001**
AR	,296**	**0,001**	,211*	**0,024**	,093	0,323	,331**	**<0,001**
DLI	**,392**	**<0,001**	,180	0,055	,081	0,388	**0,389**	**<0,001**
NS	-,118	0,210	-,131	0,163	-,066	0,481	-,159	0,090
AC	,297*	**0,001**	-,010	0,919	-,174	0,063	,152	0,104
PP	−,218*	**0,019**	,087	0,358	,100	0,286	-,097	0,302
DHLI	,401**	**<0,001**	,149	0,111	,064	0,495	,379**	**<0,001**

IS – Búsqueda de información (Information Seeking); AR – Evaluación de la fiabilidad; DLI – Determinación del nivel de interés (Determining Level of Interest); AC – Adición de contenido; NS – Habilidades de navegación (Navigating skills); PP – Protección de la privacidad; DHLI – Instrumento de alfabetización digital en salud; OSH – Búsqueda de información sanitaria en línea (Online Health Seeking); PHS – Búsqueda de información sanitaria profesional (Professional Health Seeking); THS – Búsqueda de información sanitaria tradicional (Traditional Health Seeking); HLBS – Escala de comportamientos de búsqueda de información sanitaria; r – Coeficiente de correlación de Pearson.

*p < 0,05

**p < 0,001.

Se analizaron los datos sociodemográficos, el estado de la enfermedad y las características del uso de las redes sociales de los encuestados para identificar cualquier diferencia entre las puntuaciones medias del HLBS y el DHLI, lo que reveló diferencias significativas en el nivel educativo, la frecuencia de las convulsiones y la duración del uso de las redes sociales (p < 0,05) (Figura 1).

**Figura 1:** Cambio en las puntuaciones medias del DHLI y el HLBS según el nivel educativo, la frecuencia de las crisis y el tiempo de uso de las redes sociales.

Se comparó la puntuación media del DHLI con la de la escala de nivel educativo, revelando que la puntuación media del grupo analfabeto era estadísticamente inferior a la de los demás grupos educativos. Una comparación posterior de las puntuaciones medias del Instrumento de Alfabetización Digital en Salud (DHLI) basada en la frecuencia de los turnos reveló que los participantes que realizaban pocos turnos al mes tenían puntuaciones medias significativamente más bajas que los que no trabajaban ningún turno (p < 0,05). Una comparación de la puntuación media del DHLI con el tiempo dedicado a las redes sociales reveló que quienes utilizaban las redes sociales durante 1 hora o menos al día tenían una puntuación media estadísticamente inferior a la de quienes las utilizaban entre 2 y 3 horas y 6 horas o más al día.

## DISCUSIÓN

Los resultados del presente estudio contribuyen de manera esencial al conocimiento de la relación entre los comportamientos de búsqueda de atención sanitaria y la alfabetización digital en materia de salud entre los pacientes con epilepsia, revelando los efectos de diversos factores. Los resultados sugieren que las características sociodemográficas, el estado de la enfermedad y las redes sociales pueden tener un efecto significativo en los comportamientos de búsqueda de atención médica y la alfabetización digital en salud de este grupo de pacientes.

### Características Sociodemográficas y Alfabetización Digital en Salud

Se observó que la mayoría de los participantes eran hombres, casados, graduados de secundaria, desempleados y residentes en el centro de la ciudad, y que el nivel educativo tenía un efecto significativo en la alfabetización digital en salud y los comportamientos de búsqueda de atención médica. En este sentido, el estudio respalda los hallazgos de estudios anteriores que informan de una asociación entre los altos niveles de alfabetización digital en salud y los comportamientos de búsqueda de información sanitaria en Internet entre las personas con niveles de educación más altos^(9,15)^. En el presente estudio, el hallazgo de diferencias significativas entre el nivel educativo y la alfabetización digital en salud y los comportamientos de búsqueda de atención sanitaria es coherente con hallazgos similares. En la literatura, se ha observado que, a medida que aumenta el nivel educativo, las personas son más conscientes de cómo evaluar la fiabilidad de la información sanitaria digital. En un estudio de 200 pacientes adultos mayores diagnosticados con Parkinson, se encontró que el 73% de la muestra con bajos ingresos tenía un bajo nivel de alfabetización digital en salud^(16)^. En otro estudio de adultos mayores, el 84,9% de los encuestados afirmó tener dificultades para acceder a los servicios de salud digitales, y se registró una puntuación media en la escala de alfabetización en salud electrónica de 18,43 ± 10 para la muestra^(17)^. En un estudio anterior con pacientes epilépticos, el 38,1% eran solteros y el 8,4% estaban desempleados, y se observó una correlación negativa significativa entre la residencia en zonas rurales (p < 0,001), el desempleo (p = 0,006) y el bajo nivel educativo de los pacientes (p < 0,001)^(18)^.

### Estado de la Enfermedad y Comportamiento de Búsqueda de Atención Sanitaria

El hecho de que la mayoría de los pacientes con epilepsia tomen medicación de forma regular y presenten crisis convulsivas generalizadas es otro factor crucial que afecta a los comportamientos de búsqueda de atención sanitaria. Se ha observado que las personas que viven con enfermedades crónicas como la epilepsia buscan más información sobre el manejo de la enfermedad^(10,11)^. En el presente estudio, la mayoría de los encuestados recurrieron a Internet para obtener información relacionada con la salud y recibieron tratamiento regular. Sin embargo, existe preocupación por la frecuencia con la que los pacientes buscan información sobre salud y por la fiabilidad de los materiales que encuentran. La información sobre salud a la que se accede a través de Internet varía mucho en cuanto a su precisión y fiabilidad^(8)^, por lo que no debe subestimarse la importancia de la alfabetización digital en materia de salud. Los pacientes con bajos niveles de alfabetización digital en materia de salud corren el riesgo de ser engañados por información errónea cuando buscan información sobre salud en Internet. En un estudio realizado durante la pandemia de COVID-19, un aumento de 1 punto en el acceso a la información sanitaria sobre la COVID-19 se asoció con un aumento de 3,01 puntos en la alfabetización digital en salud en general, lo que revela el grado en que las personas adoptan comportamientos de búsqueda de información sanitaria cuando enferman^(19)^.

### Uso de Las Redes Sociales y Alfabetización Digital en Salud

En el presente estudio también se evaluaron los efectos de las redes sociales en los comportamientos de búsqueda de información sanitaria. Una gran proporción de pacientes recurre a las redes sociales para obtener información. Estudios previos en la literatura señalan que las redes sociales son una fuente esencial de información sobre salud^(20, 21, 22)^. Sin embargo, cabe señalar que la información obtenida a través de las redes sociales puede ser engañosa para las personas con bajos niveles de alfabetización sanitaria^(23,24)^. En el presente estudio se encontró una asociación entre el uso de las redes sociales para obtener información y tanto la alfabetización sanitaria digital como los comportamientos de búsqueda de información sobre salud. Sin embargo, la seguridad de las redes sociales como fuente de información relacionada con la salud depende del nivel de alfabetización digital en salud de cada persona. En un estudio sobre los motivos por los que las personas utilizan Internet, las redes sociales ocuparon el primer lugar, con un 30,5%, seguidas de las videollamadas, con un 25,3%, y como fuente de datos relacionados con la salud, con un 20,2%^(17)^. En un estudio anterior con pacientes epilépticos, el 42,6% declaró conocer las aplicaciones de salud digitales^(25)^.

### Relación Entre la Alfabetización Digital en Salud y Los Comportamientos de Búsqueda de Atención Médica

Nuestros hallazgos revelan una relación débil pero significativa entre la alfabetización sanitaria y los comportamientos de búsqueda de atención sanitaria. En particular, las subdimensiones «búsqueda de información», «evaluación de la credibilidad» y «adición de contenido» de la alfabetización sanitaria digital están asociadas con los comportamientos de búsqueda de atención sanitaria. Se ha informado anteriormente de que las personas con altos niveles de alfabetización sanitaria digital son más capaces de evaluar la información sanitaria que encuentran en Internet y de actuar de forma más consciente cuando acuden a profesionales de la salud^(9)^. Por el contrario, la falta de una relación significativa entre las subdimensiones «protección de la privacidad» y «habilidades de navegación» de la alfabetización digital en salud y los comportamientos de búsqueda de atención sanitaria sugiere que las dificultades encontradas durante la búsqueda de información sanitaria en el entorno digital no están directamente relacionadas con estas habilidades. En un estudio realizado en Europa, se informó de que el 71% de los encuestados utilizaba Internet con fines relacionados con la salud, y que estos hábitos eran más frecuentes entre los jóvenes y las personas con un nivel educativo más alto, mientras que el número de visitas al médico de cabecera en el año, las enfermedades crónicas o la discapacidad y la buena valoración subjetiva de la salud también tenían un efecto positivo^(26)^. Según Eurostat, en 2020, el 55% de los encuestados en un estudio realizado en 27 países europeos utilizaba Internet para buscar información sobre salud^(27)^. Esto revela que factores socioeconómicos como el nivel de educación y los ingresos influyen en la capacidad de las personas para acceder y utilizar las herramientas digitales. De hecho, en este estudio se observó que un nivel educativo más bajo y unos ingresos más bajos estaban significativamente asociados con un menor nivel de alfabetización sanitaria (p < 0,001)^(28)^. La alfabetización sanitaria digital no solo depende de las habilidades de la persona, sino también de la accesibilidad y el diseño de las plataformas digitales. En un estudio realizado en Turquía, se observó que los encuestados diagnosticados con epilepsia tenían un nivel medio de alfabetización sanitaria, y aquellos con niveles más altos de alfabetización sanitaria tenían actitudes más positivas hacia su enfermedad^(29)^. En la bibliografía se ha descrito una asociación directa entre los bajos niveles de educación e ingresos y los niveles de alfabetización sanitaria, mientras que los niveles más altos de educación se han relacionado con una disminución de la frecuencia de las convulsiones y de las visitas a los servicios de urgencias, aunque se ha subrayado que no existe una asociación directa entre la alfabetización sanitaria y esos resultados clínicos^(28)^. Se prevé que las plataformas digitales, los materiales de información sobre medicamentos, las etiquetas, los folletos para pacientes, el apoyo del personal educativo y los resúmenes de contenidos presentados en un lenguaje sencillo y comprensible y elaborados para aumentar la alfabetización sanitaria específica sobre la epilepsia pueden contribuir a mejorar el estado de salud general de las personas al facilitar su acceso a la información sanitaria^(30)^. Este artículo sugiere que la difusión de información accesible y comprensible puede mejorar indirectamente la alfabetización sanitaria de las personas con epilepsia, así como su salud y su actitud hacia su enfermedad. Además, la inversión en alfabetización sanitaria puede desempeñar un papel estratégico en el refuerzo del control de los pacientes sobre su salud.

Este estudio ha revelado una relación bidireccional entre los comportamientos de búsqueda de atención médica de las personas con epilepsia y sus niveles de alfabetización sanitaria digital. El nivel educativo y el estado de la enfermedad, así como el uso de las redes sociales, contribuyen a la alfabetización sanitaria digital y al desarrollo de comportamientos de búsqueda de atención médica.

### Limitaciones

Las limitaciones del presente estudio incluyen su diseño unicéntrico, el pequeño tamaño de la muestra y el uso de un cuestionario para la recolección de datos. Además, la muestra solo incluyó a pacientes que acudieron al hospital, por lo que no fue posible contactar con un grupo de pacientes más amplio. En futuros estudios se puede examinar esta cuestión con mayor profundidad mediante la participación de una muestra más amplia y diferentes métodos de investigación.

## CONCLUSIÓN

Este estudio ha revelado una asociación débil pero significativa entre la alfabetización digital en salud y los comportamientos de búsqueda de atención médica en pacientes con epilepsia, en los que un bajo nivel educativo, unos ingresos elevados y un uso limitado de las redes sociales se asocian con niveles reducidos de alfabetización digital en salud y hábitos de búsqueda de atención médica. Estos hallazgos sugieren que es necesario desarrollar programas educativos específicos destinados a aumentar la alfabetización sanitaria digital en este grupo de pacientes, y que los profesionales sanitarios reciban formación sobre cómo contribuir a la disponibilidad de información sanitaria precisa y fiable en el entorno digital. Si se orienta a los profesionales sanitarios para que compartan información sanitaria precisa con datos sobre el envejecimiento digital, se facilitará el acceso a características precisas de los pacientes.

## Data Availability

Los datos que respaldan los hallazgos de este estudio están disponibles a través del autor de correspondencia previa solicitud razonable.
